# Penetrating Offenders in Hemifacial Spasm: Surgical Tactics and Prognosis

**DOI:** 10.3390/life13102021

**Published:** 2023-10-07

**Authors:** Hyun-Seok Lee, Kwan Park

**Affiliations:** 1 Department of Neurosurgery, Konkuk University Medical Center, Seoul 05030, Republic of Korea; 20220205@kuh.ac.kr; 2 Department of Neurosurgery, Sungkyunkwan University School of Medicine, Seoul 06351, Republic of Korea

**Keywords:** hemifacial spasm, microvascular decompression, penetrating offender

## Abstract

(1) Background: In cases of hemifacial spasm (HFS), there are various patterns related to the vascular compression of the facial nerve, including a very rare form that is seen when the offending vessel penetrates the facial nerve. However, there have been few reports in the literature regarding the associated surgical techniques and postoperative prognosis. (2) Methods: A retrospective review was conducted of 4755 patients who underwent microvascular decompression (MVD) surgery from April 1997 to June 2023. In total, 8 out of the 4755 patients (0.2%) exhibited a penetrating offending vessel; the medical and surgical records of these 8 patients were then analyzed. Surgery was then attempted to maximally decompress the penetrating offender. (3) Results: Seven out of the eight patients (87.5%) were spasm-free immediately after surgery, and one had only 10% residual spasm compared to their preoperative condition. That patient was also spasm-free one year later. Postoperative facial palsy occurred in one patient (12.5%) who was assessed as grade II in the House–Brackmann grading system. In another patient, the resection of a small facial nerve bundle did not result in facial palsy. There were no cases of hearing loss or other complications. (4) Conclusions: Decompressing the penetrating offender did not increase the incidence of facial palsy, and the prognosis for hemifacial spasms was good. Therefore, when a penetrating pattern was encountered during MVD surgery, decompression between the penetrating offender and the facial nerve may offer good results.

## 1. Introduction

Hemifacial spasm (HFS) is a form of neurovascular syndrome that is usually due to neurovascular compression in the root exit zone (REZ) of the facial nerve. The disease presents as an intermittent, involuntary facial twitching movement that usually begins in the eyelids and progresses to involve the entire system of ipsilateral facial muscles. The result is an asymmetrical appearance of the face, due to the strengthening of the facial muscles on the side of the spasm. The pathogenesis of HFS is thought to be derived from the vascular compression of the facial nerve that emerges close to the brain stem, leading to demyelination and ephaptic transmissions [1,2].

There are various treatments for HFS, such as medications and *Botulinum toxin* injections, but, compared to these treatments, MVD surgery is the most effective treatment and one that completely resolves the symptoms [3,4,5,6,7,8,9]. The overall rate of being spasm-free after MVD surgery in patients with HFS is approximately 90%, with the other 10% of patients experiencing a recurrence of facial spasm or surgical failure [3,10,11]. In the 10% of cases where this facial spasm did not resolve itself, in rare instances, the surgeons may have encountered unusual patterns of compression that would make for a very difficult surgical challenge. In 2007, we categorized six different patterns of facial nerve compression, describing their clinical implications and prognosis [12]. As the number of cases increased, we began to see other rare and difficult cases [11], one of which was the perforating pattern, where the offending vessel penetrated the facial nerve.

There are currently no reports as to how this pattern should be decompressed, whether complications such as postoperative facial palsy and hearing loss after decompression are likely, and with what factors they are associated; this is the focus of the current study.

## 2. Materials and Methods

### 2.1. Patient Cohort

We retrospectively analyzed the medical records of 4755 patients who underwent MVD with HFS from April 1997 to June 2023. All MVD surgeries were performed by a single surgeon (Kwan Park), with facial nerve motor-evoked potential (facial MEP), lateral spread response (LSR), and brain stem auditory evoked potential (BAEP) being monitored by an experienced neurophysiologist. The clinical information and details of the offending vessels of all 4755 patients are summarized in Table 1. Cases with 2 or 3 offending vessels present a sandwich pattern or a tandem pattern, in which the vessels are compressed together.

Of these 4755 patients, 8 exhibited the penetrating type of pattern (0.2%). All penetrating offenders were confirmed via surgical microscopic findings. Of these 8 patients, 4 were male, 4 were female, 5 had right-sided lesions, and 3 had left-sided lesions. The median age of the patients was 41.5 years (an age range of 23–67). Of the 8 patients, the two oldest patients (67 and 55 years old) had underlying hypertension and were on anti-hypertensive medication, and the next-oldest patient (49 years old) was taking medication for diabetes mellitus. The other 5 patients had no underlying medical conditions.

The degree of preoperative facial spasm was analyzed using our previously published SMC grading system [13,14]. Grade I refers to situations when the spasm is localized to the periocular area, whereas grade II refers to situations when the involuntary movement spreads to other areas of the ipsilateral face and affects other muscles, such as the orbicularis oculi, frontalis, zygomaticus, mentalis, and platysma. Grade III represents the disruption of vision due to frequent spasms, whereas grade IV refers to a persistent spasm resulting in significant facial asymmetry. Among the 8 patients under study, the degree of preoperative spasm was classified as grade II in 3 patients (37.5%), as grade III in 3 patients (37.5%), and as grade IV in 1 patient (12.5%), whereas the grade of the final patient could not be determined.

The mean duration of postoperative spasm symptoms was 60.4 months (in a range of 18–120 months), with 4 patients being prescribed medication for preoperative treatment, 4 patients receiving botulinum toxin (Botox) injections, and 2 patients receiving both Botox injections and medication. One patient was prescribed medication and acupuncture at an oriental medicine clinic, whereas another received botulinum toxin treatment and acupuncture at an oriental medicine clinic. The final 2 patients received no other treatment prior to MVD surgery (see Table 2).

### 2.2. Operative Technique and Intraoperative Monitoring

The patients underwent MVD surgery via a retromastoid suboccipital craniotomy (RMSOC) while in the park bench lateral position. All patients underwent MVD surgery with intraoperative neuromonitoring, which consists of a real-time BAEP monitoring method [14] and the facial motor evoke potential (fMEP) and lateral spread response (LSR), i.e., abnormal muscle response (AMR). The evaluations of intraoperative LSR disappearance were categorized as follows: disappearance after durotomy and cerebrospinal fluid drain from the lateral medullary cistern; disappearance after immediate decompression, where the amplitude of LSR decreases but does not disappear completely; and no disappearance. In all patients, decompression of the neurovascular conflict zone was performed using Teflon felt.

### 2.3. Assessment of Postoperative Outcomes

All patients underwent preoperative intra-auditory canal (IAC) magnetic resonance imaging (MRI) to identify the most likely offender, the results of which were reviewed by an experienced neuroradiologist. Preoperative audiometry, including pure tone audiometry (PTA) and speech audiometry (SA), was performed, and the patients also underwent preoperative lateral spread response (LSR) testing conducted by an experienced neurophysiologist. Computed tomography (CT) of the brain was performed immediately after the operation in all patients, and a temporal bone CT scan was also taken postoperatively on day three in all patients. Postoperative pure tone audiometry (PTA) and SA (speech audiometry) was performed postoperatively on days 4–5, and the results were then compared with the preoperative results. The patient’s spasms were assessed preoperatively, immediately after surgery (until 5 days after surgery), 1 month after surgery, 1 year after surgery, and up to 2 years after surgery. The lateral spread response (LSR) was examined just before the patient’s outpatient department visit after their discharge.

## 3. Results

In the eight patients identified, the offending vessels were as follows: the anterior inferior cerebellar artery (AICA) in six patients (75%), the branch of the AICA in one patient (12.5%), and the posterior inferior cerebellar artery (PICA) in one patient (12.5%). The intraoperative LSR disappearance pattern was IIa (i.e., the vessel disappeared immediately after decompression) in seven patients (87.5%) and IIc (i.e., a 50% reduction with a residual presence) in one patient (12.5%).

An intraoperative change in BAEP was seen in three patients. Two had a 50% decrease in amplitude, prolonged by 1.6 ms and 2.0 ms, respectively, with full recovery by the end of surgery, and one exhibited the loss of all but wave I, with 80% recovery by the end of surgery. There was no long-term hearing loss after surgery. It is worth noting that three of the eight patients underwent surgery before our real-time BAEP monitoring method was established. All three exhibited no postoperative hearing loss.

Postoperative facial palsy was seen in one patient (12.5%). Immediately after surgery, the palsy was classified as House–Brackmann grade III–IV, and the patient showed gradual improvement to grade II but still demonstrated residual issues. The other seven patients (87.5%) exhibited no facial palsy.

The prognosis of postoperative spasm was evaluated immediately after surgery (around 5 days), 1 month after surgery, 1 year after surgery, and 2 years after surgery. In seven of the eight patients (87.5%), there was no spasm immediately after surgery. One patient had 10% residual spasms compared to their preoperative state. At 1 month after surgery, one of the seven patients who had no spasm immediately after surgery had 10% residual spasm compared to their preoperative state, and the one patient with 10% residual spasm had worsened slightly to 20%. At 1 year after surgery, one patient with a new 10% spasm at 1 month had similar symptoms, and one patient with symptoms shortly after surgery was then spasm-free. Of the other six patients who were asymptomatic immediately after surgery, three remained spasm-free, one patient was lost to follow-up, one patient with no spasm developed a spasm of approximately 10% compared to preoperative levels, and one had not yet reached the one-year postoperative mark. Upon follow-up in year 2, two patients with a residual 10% of spasms at 1 year after surgery were completely spasm-free after 2 years, and one patient who had spasms immediately after surgery and after 1 month remained spasm-free at 1 and 2 years after surgery. Of the three patients who were followed up, one was lost to follow-up, and two continued to be spasm-free (one of these two is a patient who developed facial palsy after MVD surgery) (see Table 3).

### Illustrative Case

**Figure 1:**
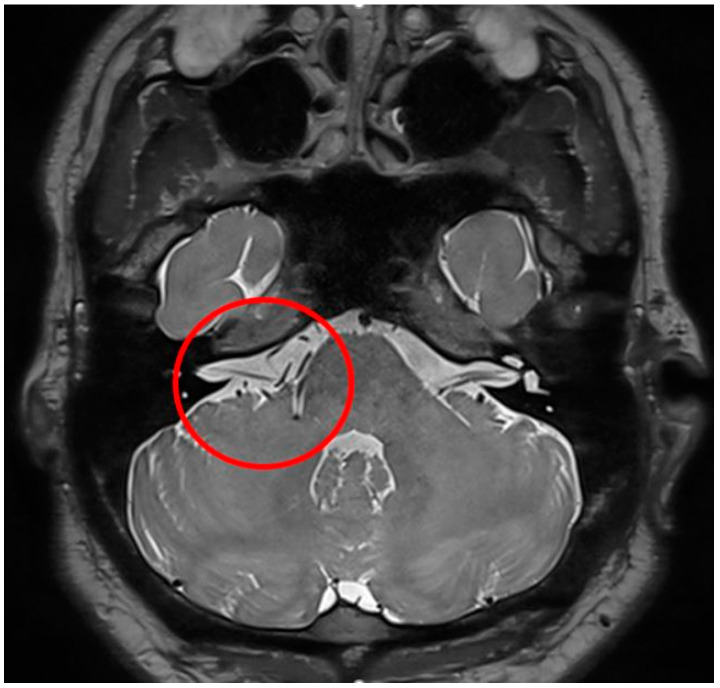
A 34-year-old man with right HFS, shown in a preoperative proton density-weighted (PD-weighted) magnetic resonance image (MRI). At the exit region of the right facial nerve root, the right anterior inferior cerebellar artery (AICA) and posterior inferior cerebellar artery (PICA) have a complex appearance (red circle).

**Figure 2:**
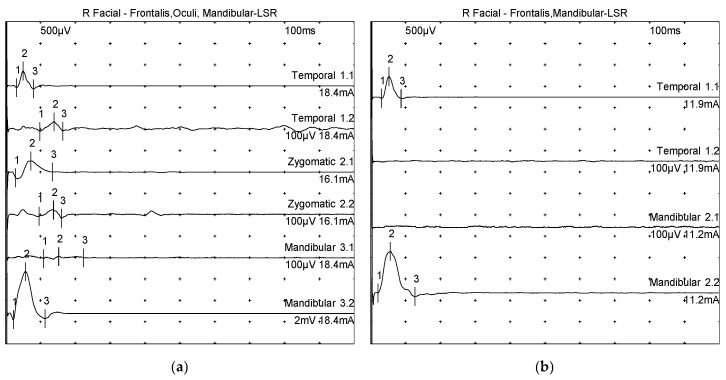
Right-side facial nerve conduction studies of a patient acting as an illustrative case. In both picture, each number is a reference point for measurement. ‘1’ is onset latency, from ‘1’ to ‘2’ is onset to peak amplitude, and from ‘2’ to ‘3’ is peak to peak amplitude: (**a**) the preoperative examination showed lateral spread response (LSR); (**b**) in the postoperative examination, the LSR seen before surgery has disappeared.

A 34-year-old man developed twitching of the right eye and mouth two and a half years prior to admission and was subsequently diagnosed with HFS. After medication and botulinum toxin treatments, he was referred for MVD surgery and visited our institution. His pre-operative spasm grade was SMC grade II; magnetic resonance imaging showed a complex REZ, which the radiologist interpreted as demonstrating that both the AICA and PICA were in contact in the REZ (Figure 1). LSR was observed in the right facial nerve during an examination with electrical stimulation (Figure 2a).

**Figure 3:**
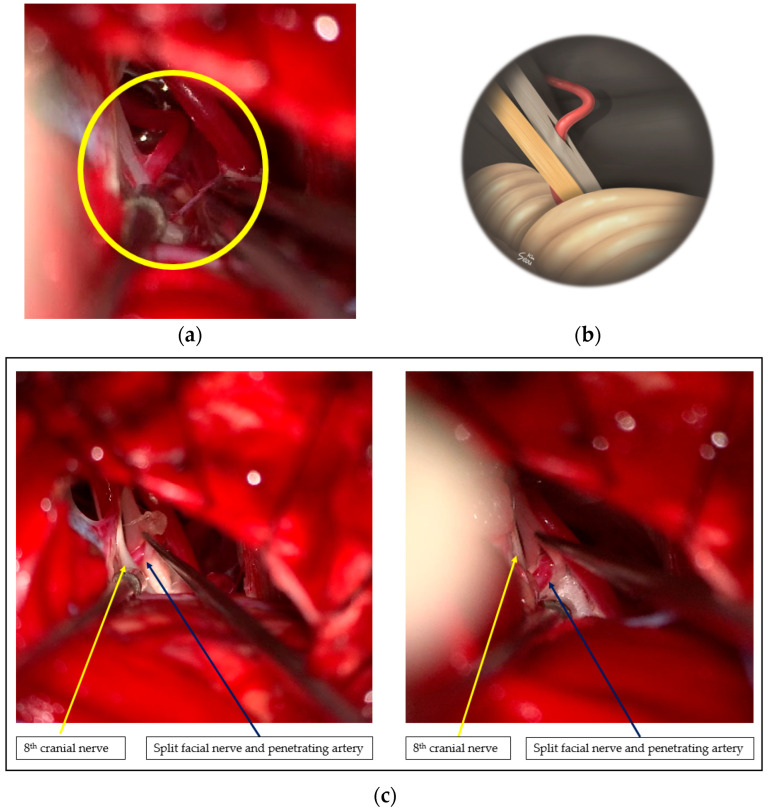
Intraoperative microscopic findings for the illustrative case: (**a**) a branch of the anterior inferior cerebellar artery (AICA) passing through the facial nerve, with no other indentations visible in the root exit zone (yellow circle); (**b**) illustration of the surgical visualization before decompression. The illustration shows the offending artery passing through the facial nerve; (**c**) Microscopic findings with decompression in progress on both the left and right sides. The yellow arrow is the 8th cranial nerve, and the navy arrow shows a branch of the AICA penetrating the facial nerve, both images.

Intraoperatively, the offending vessel was a branch of the AICA, which was penetrating the facial nerve (Figure 3a–c).

**Figure 4:**
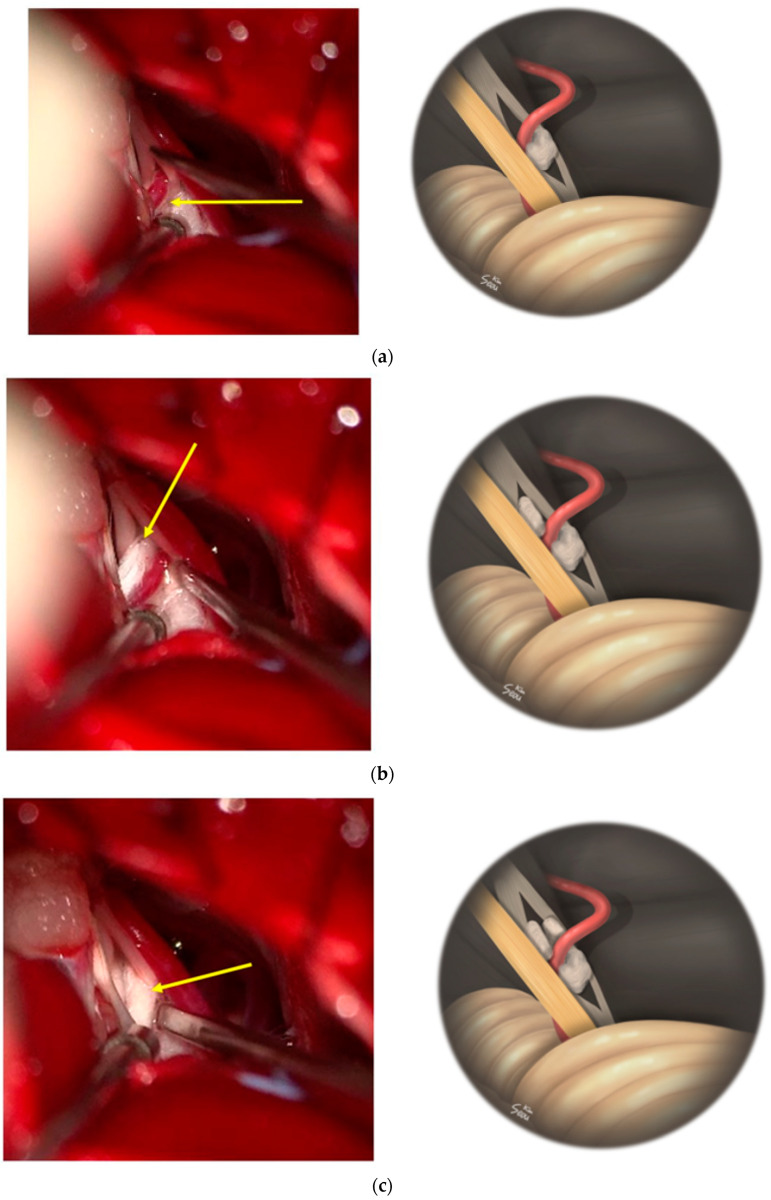
(**a**) First, decompression was started with small Teflon felt, from inferior side of the perforation site (yellow arrow); (**b**) Next, decompression was performed in supero-medial (supero-anterior) side (yellow arrow); (**c**) Lastly, the supero-lateral (supero-posterior) side decompression was performed (yellow arrow) to decompress in all directions of the perforation site, and the lateral spread response (LSR) subsequently disappeared.

First, we checked whether there was a space between the facial nerve and the penetrating artery and carefully dissected the facial nerve and the penetrating branch of the AICA to decompress in all directions (Figure 4a–c).

Immediately after decompression, the LSR disappeared, and the surgery was completed. The patient in question had no spasms and no facial palsy immediately after surgery and was discharged on day 5 with no further complications. Postoperative audiometry showed no hearing difficulty. One month after surgery, the patient still had no spasms, and the disappearance of LSR was confirmed via a postoperative neurophysiological study (Figure 2b).

## 4. Discussion

In 2008, we analyzed 236 cases of HFS, reporting several patterns of neurovascular compression of the facial nerve [12]. At the time, we categorized the patterns into six types: arachnoid (28.0%), loop (4.7%), and perforator (24.6%), which are generally compressed by a single causative vessel, and branch (7.6%), sandwich (11.9%), and tandem (22.0%), which are compressed by two or more vessels [12]. There were also three cases (1.3%) in which the pattern category was not clear at the time. As the number of surgeries grew, we added new neurovascular compression patterns. One is the encircling pattern, in which the vessel encircles the facial nerve in a 270-degree or even 360-degree loop. Another is the penetrating type, in which the culprit vessel passes completely through the facial nerve [11]. These patterns of neurovascular compression are particularly challenging to operate on, especially the penetrating type, as they are often not clearly identifiable from a magnetic resonance image, and cases are so rare that there are no established decompression methods.

There is a very limited corpus of literature on HFS caused by a penetrating offender; however, a report was published for a single case in 2015. Oh et al. reported a left HFS presenting in a 20-year-old male patient. The penetrating offender in this patient was the AICA; after decompression, the spasm disappeared, but definite facial palsy occurred [15]. From this case report and from our study, it is apparent that HFS caused by the penetrating type occurs at a relatively young age compared to the general population. Typically, HFS is reported to be more prevalent in those in their 40s and 50s [16,17]. Similarly, when analyzing all 4755 of our cases, the median patient age was 53 years old (range: 17–75), and the median age of the eight patients with the penetrating type was 41.5 years (range: 23–67). This has the clinical implication that symptoms occur at a younger age in the case of penetrating offenders (Table 4). It is conceivable that the symptoms occur at a younger age because the causative vessels (mostly the AICA) directly irritate the facial nerve. It is also possible that if the facial nerve is separated, myelination is less well developed than if it is not separated. Reports of duplication of the facial nerve in the mastoid segment or its distal part are rare in the ENT department [18,19,20], but there are few reports of duplication in the intracranial portion from the root exit zone of the facial nerve to the entry point of the IAC; therefore, further anatomic and pathologic studies are required.

In general, the same trend is seen in our 4755 cases; the AICA is the most common offending vessel for HFS, whereas the second most common is the PICA, followed by the AICA and other vessel compressions in conjunction (see Table 1) [12,21]. The PICA originates in the VA and emerges from the ventral to the dorsal side of the brain stem, usually at the level of the lower cranial nerves (at the 9th, 10th, and 11th cranial nerves), forming a hairpin structure (caudal loop) downward [21,22]. Therefore, while it is possible for a vessel to loop and compress the root exit zone of the facial nerve or encircle the facial nerve, it is structurally difficult for the vessel to pass through the facial nerve. In the cases examined in this study, only one out of the eight (12.5%) showed penetration by the PICA, and almost all of them showed penetration by the AICA (87.5%) (see Table 3). The facioauditory primordium, the origin of the facial nerve, appears in the third week of gestation. It then splits into two sections at the end of the fourth week and is complete by the fifth or sixth week of gestation [15,23]. The AICA begins to develop later, in the gestational fourth or fifth month, when the facial nerve is already fairly well-developed [15,24]. Therefore, facial nerve penetration by the artery is thought to occur as the facial nerve attempts to pass through the divergence that appears after 5–6 weeks of gestation.

In all patients, we attempted complete decompression in all directions and a 360-degree inspection in all patients, as in the illustrative case. First, check for space in the perforating artery and facial nerves with a micro-dissector or micro-bayonet forceps. Then, carefully dissect, and, when space is made, put small Teflon felt in there to first decompress. Next, we decompressed in all possible directions (Figure 4a–c). Decompressing the location between the facial nerve and penetrating vessels was associated with a very low probability of postoperative facial palsy. In one case, we decompressed only the medial side in a patient presenting a difficult dissection. As the LSR improved when the medial side was decompressed in this patient, the lateral side, which was difficult to meticulous dissection and presented a high risk of facial nerve injury upon dissection, was left untreated, and the operation was terminated. Postoperatively, the facial spasm had been resolved. In another case, the fascicle of the facial nerves was divided into 90% and 10% divisions by a penetrating PICA. In this patient, decompression of the thin side of the divided nerve fascicle was not possible. We first stimulated the thin nerve fascicle with a direct nerve stimulator to check the facial muscle response, which showed a small response by the orbicularis oris muscle. The thin fascicle that could not be decompressed and dissected was then excised, and the neurovascular compression between the large fascicle and the offending vessel was decompressed with Teflon felt. Immediately after decompression, the LSR disappeared. After the surgery, facial spasms remained at 10–20% compared with preoperative conditions; notably, there was no postoperative facial palsy. The absence of facial palsy despite the resection of a portion of the facial nerve in this patient was thought to be related to the innervation of the distal branch of the facial nerve. The facial nerve has five branches, namely, the temporal, zygomatic, buccal, marginal, and cervical branches, each of which is known to have interconnections, and these connections vary considerably [25,26,27,28]. In addition, several studies have shown that facial nerve is anatomically connected to the trigeminal nerve [25,27,28,29,30,31], vestibulocochlear nerve [25,32], glossopharyngeal nerve [25,33], and vagus nerve [25], as well as the cervical plexus [25,34]. In particular, there are numerous interconnections and variations between the zygomatic and buccal branches of the facial nerve, which can confound LSR measurements; we have previously published a paper on how to measure LSR in this context [26]. As the orbicularis oris muscle is often innervated by both the zygomatic and buccal branches, we consider that facial palsy did not occur despite the partial resection of the nerve fascicle during the operation. Maximal decompression using Teflon felt placed between the penetrating vessel and the facial nerve was performed in all eight patients, as mentioned, and attempted decompression in all compression sites. One patient developed postoperative facial palsy that was classified as House–Brackmann grade II, but the other seven patients exhibited no facial palsy and had good prognoses regarding postoperative facial spasm (see Table 3). Therefore, even if the artery were going through the facial nerve, it is considered necessary to actively dissect and decompress it. Yet, the most important thing we and other very experienced surgeons of more than 4000 cases emphasize is to check the neurovascular conflict completely, it is important to check the full inspection of facial nerve and entire 360 degrees of REZ if possible [35]. Also, even if you encounter a perforating artery, you should look for offenders in the REZ. This is because MVD of HFS is not an emergency surgery, but a functional neurosurgery, and it is recommended to decompress all offenders safely.

There is also a nervus intermedius that can be confused with a split in the facial nerve. The nervus intermedius was first identified in 1563; it was first named “portio media intercommunicantem faciei et nervum auditorium” in 1777 by Heinrich August Wrisberg [36,37]. The nervus intermedius is thus named because it is located between the facial nerve and the superior portion of the vestibular cochlear nerve [37,38]. The nervus intermedius carries parasympathetic nerve fibers to the nasopalatine and lacrimal glands, conducting sensory information from facial areas such as the concha of the ear and the nose [37,38,39,40,41]. Irritation of the nervus intermedius can cause geniculate neuralgia, which is expressed as intermittent but severe sharp pains deep in the ear, accompanied by the disruption of salivation, the sense of taste, and lacrimation [42]. Geniculate neuralgia can also be caused by neurovascular compression, which is an indicator of the need for MVD surgery. Lovely and Jannetta reported on 14 cases of MVD surgery in patients with geniculate neuralgia [43]. In their paper, they reported that vascular compression of the nervus intermedius was present in the surgical findings and correlated with symptoms such as deep ear pain [43]. In surgical findings that appear to show a perforating offender, it is possible to differentiate between the perforating offender and the nervus intermedius by tracing the entire course of the facial nerve. It may be helpful to perform direct nerve stimulation to check the response of the facial muscles. As mentioned above, the nervus intermedius is mainly a sensory nerve; when stimulated, it manifests as ear pain [38], rather than as a facial muscle response. It is also thought to be helpful in the differentiation of preoperative symptoms.

A limitation of this study is the small cohort of eight cases. This refers to 8 out of 4755 cases, so a very small probability of 0.2%. This means that the chances of actually encountering a perforating offender during MVD surgery are very low. In addition, this study was based on data regarding the experiences of a single surgeon, and it was not possible to compare the findings with results from other institutions. This is because there are few reports regarding HFS with a perforating offender that are available from other institutions. However, this limitation also means that our surgical method can act as a reference for the operation. As more data are accumulated from continuing MVD surgeries, in the future, we expect to analyze the prognoses and casual factors in a large series.

## 5. Conclusions

In as little as 0.2% of the 4755 cases examined in this study, offenders of the penetrating type are very rare, but they do exist. Cases of penetrating offenders in the context of HFS have been reported very rarely, and there is no established policy for decompression. In our experience, when the penetrating offender and the facial nerve fascicle are delicately dissected and decompressed using a small piece of Teflon felt, the facial spasm is resolved, and the likelihood of postoperative facial palsy is very low. In the case where it was necessary to cut a minor portion of a facial nerve that was difficult to decompress, it was found that the patient recovered without exhibiting facial palsy.

The statistical analysis of the factors involved showed that such patients were treated at a relatively young age compared to the general age of HFS patients. For this reason, in HFS cases at a relatively young age, when the offending vessels in the root exit zone are unclear on an MRI scan, it is advisable to consider the possibility that the offending vessel is of the penetrating type. In addition, if encountering a penetrating offender during MVD surgery, it is recommended that the surgeon should not hesitate but instead conduct careful dissection and complete decompression.

## Figures and Tables

**Table 1:** Clinical characteristics of 4755 patients treated with MVD surgery for hemifacial spasm.

Clinical Characteristics	
Median age at MVD (range (years))	53 (17–75)
Sex (male:female)	1395: 3360
Operation side (left:right)	2441: 2314
Median duration of symptom (in months)	48
Average length of hospital stays, in days (range)	7.4 (3–185)
Penetrating offender type (%)	8 (0.2%)
**Offending vessel, *n* (%)**	
AICA	2635 (55.4%)
PICA	1011 (21.3%)
AICA-PICA common trunk	14 (0.3%)
VA	50 (1.1%)
AICA + PICA	275 (5.8%)
AICA + VA	469 (9.9%)
PICA + VA	162 (3.4%)
AICA + vein	21 (0.4%)
PICA + vein	5 (0.1%)
AICA + PICA + VA	61 (1.3%)
Vein only	44 (0.9%)
Other vessels	5 (0.1%)
Cannot be confirmed	3 (0.0%)

MVD: microvascular decompression, AICA: anterior inferior cerebellar artery, PICA: posterior inferior cerebellar artery, VA: vertebral artery.

**Table 2:** Clinical characteristics of 8 patients with penetrating offenders.

No.	Sex	Age	Laterality	Preoperative Spasm Grade	Preoperative Treatment	Symptom Duration (Month)	Medical History
1	M	55	R	N/A	None	120	HTN
2	M	67	R	IV	Medication, Oriental Medicine	84	HTN
3	F	49	L	II	BTX injection, Oriental Medicine	48	DM
4	F	44	L	III	BTX injection	18	None
5	F	39	L	III	Medication	78	None
6	M	23	R	II	None	46	None
7	F	36	R	III	Medication, BTX injection	61	None
8	M	34	R	II	Medication, BTX injection	28	None

No: number, M: male; F: female, R: right, L: left, BTX: botulinum toxin, HTN: hypertension, DM: diabetes mellitus, N/A: not available or not mentioned.

**Table 3:** Intraoperative findings and prognosis of 8 patients with penetrating offenders.

No.	Offender	LSR Disappearance	Intraoperative BAEP Change	Postoperative Spasm (%)				Postoperative Facial Palsy	Surgical Findings
				~5D	~1M	~1Y	~2Y		
1	AICA	Disappeared after decompression	No change	0	0	0	0	H-B II	N/A
2	AICA	Disappeared after decompression	No change	0	0	N/A	N/A	None	N/A
3	AICA	Disappeared after decompression	No change	0	0	0	0	None	N/A
4	AICA	Disappeared after decompression	50% decreased, delay to 2.0 ms, full recovery	0	10	10	0	None	Only the medial side was decompressed because the lateral side was difficult to decompress; LSR disappearance after decompression
5	PICA	Disappeared after decompression	All but wave I disappeared, recover to 80%	10	20	0	0	None	90% nerve fascicle on the medial side of the PICA, and 10% on the lateral side, with adhesion; therefore, a minor portion was resected after detachment. Weak response from the orbicularis oris when stimulated before resection.
6	AICA	Decline amplitude, but not disappeared	50% decreased, delay to 1.6 ms, full recovery	0	0	0	N/A	None	On the lateral side, the AICA circled between CNs 7 and 8, penetrated the facial nerve medially, and exited laterally.
7	AICA	Disappeared after decompression	No change	0	0	10	0	None	The facial nerve was split, and the AICA passed through the center; a small piece of Teflon felt was placed inside the split to decompress the nerve.
8	Branch of AICA	Disappeared after decompression	No change	0	0	N/A	N/A	None	LSR disappeared when decompressing the penetrating vessel.

No: number, LSR: lateral spread response, BAEP: brain stem auditory evoked potential, D: day, M: month, Y: year, AICA: anterior inferior cerebellar artery, PICA: posterior inferior cerebellar artery, N/A: not available or not mentioned, H-B: House–Brackmann grade, CN: cranial nerve.

**Table 4:** Factors correlating with penetrating offenders and hemifacial spasm.

Factor	Univariate (*p*-Values)	Multivariate (*p*-Values)
Sex (female)	0.213	0.258
Age	0.038	0.052
Lesion side (Right)	0.439	0.435
Symptom duration	1.000	0.621
Hospital days	0.808	0.694

## Data Availability

All data included in this study can be provided by contacting hs5937@hanmail.net.
